# Adaptive Synchronization Strategy between Two Autonomous Dissipative Chaotic Systems Using Fractional-Order Mittag–Leffler Stability

**DOI:** 10.3390/e21040383

**Published:** 2019-04-10

**Authors:** Licai Liu, Chuanhong Du, Xiefu Zhang, Jian Li, Shuaishuai Shi

**Affiliations:** 1 School of Electronic and Information Engineering, Anshun University, Anshun 561000, China; 2 School of Mathematics and Computer Science, Guizhou Education University, Guiyang 550018, China; 3 School of Information Engineering, Guizhou University of Engineering Science, Bijie 551700, China

**Keywords:** fractional-order, chaotic system, Mittag–Leffler stability, adaptive laws

## Abstract

Compared with fractional-order chaotic systems with a large number of dimensions, three-dimensional or integer-order chaotic systems exhibit low complexity. In this paper, two novel four-dimensional, continuous, fractional-order, autonomous, and dissipative chaotic system models with higher complexity are revised. Numerical simulation of the two systems was used to verify that the two new fractional-order chaotic systems exhibit very rich dynamic behavior. Moreover, the synchronization method for fractional-order chaotic systems is also an issue that demands attention. In order to apply the Lyapunov stability theory, it is often necessary to design complicated functions to achieve the synchronization of fractional-order systems. Based on the fractional Mittag–Leffler stability theory, an adaptive, large-scale, and asymptotic synchronization control method is studied in this paper. The proposed scheme realizes the synchronization of two different fractional-order chaotic systems under the conditions of determined parameters and uncertain parameters. The synchronization theory and its proof are given in this paper. Finally, the model simulation results prove that the designed adaptive controller has good reliability, which contributes to the theoretical research into, and practical engineering applications of, chaos.

## 1. Introduction

Research on chaotic systems has not ceased over the past fifty years. Scholars have discovered a large number of three-dimensional integer-order chaotic systems, such as the Lorenz system [1,2,3], the Chen system [4], the Liu system [5], and the Rossler system [6]. Subsequently, on the basis of these classical systems, novel variations on chaotic systems have been studied [7,8,9,10]. Due to their very rich dynamics and complex characteristics, these nonlinear chaotic systems have been applied in many fields, such as meteorology [11,12], mechanics [13,14,15], and secure communication [16,17,18,19].

Recently, much research has been done on fractional calculus theory. It has been found that if a system is described using a fractional order, we can more effectively discover its behavior and characteristics. The same is true for a nonlinear chaotic system with a fractional order. Many researchers, therefore, have devoted themselves to research in this field. For example, Junguo Lu proposed the fractional Lü system and a synchronization method for it [20]. Shaobo He and Kehui Sun proposed a generalized fractional synchronization theory and a DSP implementation for it [21]. Marius-F. Danca studied the chaotic characteristics of hidden attractors for generalized fractional-order Lorentz systems, fractional-order Rabinovich–Fabrikant systems, and nonsmooth fractional-order Chua systems [22]. It is worth pointing out that these studies are primarily directed at three-dimensional fractional-order chaotic systems. Fractional-order chaotic systems with a higher number of dimensions have advantages in terms of nonlinear complexity, and there are still many unknown features to discover. However, there is a paucity of studies that focus specifically on multivariable chaotic systems. It is necessary to conduct further research into fractional-order systems with a high number of dimensions.

It has been found that fractional-order chaotic systems have higher nonlinearity and a higher spreading power spectrum compared to integral ones [23]. So, these systems have a very broad range of potential applications in the field of secure communication and other related sciences where chaotic synchronization is the key technology. Scholars in nonlinear control disciplines have proposed a number of effective synchronization methods since the pioneering work of Pecora and Carroll in 1996 [24], including drive-response synchronization [25], active–passive synchronization [26], coupled synchronization [27], continuous variable feedback synchronization [28], adaptive synchronization [29,30,31,32,33], pulse synchronization [34], projection synchronization [35], finite time synchronization [36], sliding mode control synchronization [37], hybrid synchronization [38,39], and other methods [40,41,42]. Chaotic synchronization is a kind of chaos control technology. The adaptive synchronization method has mostly been applied to integer-order chaotic systems. For example, A Khan and A Tyagi studied a new adaptive control method for hyperchaotic systems with unknown parameters according to the Lyapunov stability theory [38]. Feki Moez combined the Lyapunov stability theory with an adaptive law to realize the synchronization of an integer-order Lorenz system and applied it to secure communication [31]. S. Vaidyanathan et al. implemented adaptive synchronization of the same structure according to the Lyapunov theory into a conservative chaotic system with three-dimensional hidden attractors [30]. The reason why adaptive synchronization is usually used for integer-order chaotic systems is that it is convenient when computing the integer-order derivative for the Lyapunov function constructed using the system error. In contrast, for a fractional-order system, since the synchronization error function is also fractional, the fractional-order terms of the Lyapunov function are difficult to process, which can only be solved by seeking an inequality substitution [43,44] or by designing a sliding mode control surface [37,45,46,47]. A more general adaptive synchronization methodology for fractional-order chaotic systems does not exist at present.

Motivated by the above discussion, this paper investigates two novel four-dimensional fractional-order chaotic systems with different complexities, and establishes a universal adaptive law based on the Mittag–Leffler [48,49] fractional stability theory. First, in order to improve the dynamic behavior of nonlinear systems and the security of communication systems, two four-dimensional, fractional-order, and nonlinear chaotic systems are designed. The two systems have different numbers of equilibrium points. Their nonlinear dynamic behavior and complex dynamics are analyzed by means of phase diagram, time domain diagram, bifurcation diagram, power spectral density, and information entropy analysis. After that, instead of adopting the conventional method of constructing an adaptive law by constructing a complicated function in order to use the Lyapunov stability theorem, an adaptive controller was realized based on the fractional-order Mittag–Leffler stability theory. Irrespective of whether or not the parameters change, the adaptive control theory is used to synchronize two chaotic systems, which greatly enriches and extends the theoretical method for fractional-order synchronous control with different structures.

The rest of this paper is organized as follows. Section 2 defines the fractional calculus and the Mittag–Leffler lemma, gives the mathematical models of the two four-dimensional fractional-order chaotic systems, and presents the numerical simulation of the nonlinear dynamics. The complexity $C_{O}$ of the two systems is analyzed from the perspective of the number of simulation points N and the system parameter values in Section 3. In Section 4, an adaptive law for the synchronization of fractional-order systems is proposed for the cases of determined parameters and uncertain parameters. Section 5 summarizes the conclusions.

## 2. Fractional-Order Chaotic System Description and Chaotic Behavior Analysis

### 2.1. The Fractional Calculus and the Mittag–Leffler Stability Theorem

There are several ways to define the fractional calculus, including the Riemann–Liouville (R-L) definition [50,51], the Caputo definition [52], and the Grünwald–Letnikov (G-L) definition [53]. Among them, the R-L definition and the Caputo definition are the most commonly used. Compared to the other two definitions, the Caputo definition is more widely used in the engineering field. It is suitable for the description of an initial value problem in fractional differential equations. Therefore, we choose the Caputo definition.

**Definition** **1.** *The Caputo fractional differential is defined as*

(1) $${}_{a}^{C}D_{t}^{q}f(t)=\frac{1}{\Gamma (n-q)}\times \int_{a}^{t}\frac{f^{\left(n\right)}(\tau )}{{\left(t-\tau \right)}^{q-n+1}}d\tau$$

*where C indicates that this definition is the Caputo fractional-order definition, q is the order of the differential calculus, n is the smallest integer greater than q, and t and a are the upper and lower limits of the definite integral, respectively.* Γ(⋅) *is the Gamma function.*

**Lemma** **1.** *Properties of the Caputo Derivative Operator* [54]

*If*x(t)∈R*is a continuous differentiable function, then, for any*t≥b, the following relation holds:

(2) $$\frac{1}{2}{}_{b}^{C}D_{t}^{\alpha }x^{2}(t)\leq x(t){}_{b}^{C}D_{t}^{\alpha }x(t),\;\forall \alpha \in (0,1)$$

**Definition** **2.** *Consider the Caputo fractional nonautonomous system*

(3) $${}_{a}^{C}D_{t}^{q}x(t)=f\left(t,x\right)$$

*with initial condition*x(a)*, where*0<q<1*,*$f:\left[a,\infty \right]\times \Omega \to R^{n}$*is piecewise continuous in*t*and locally Lipschitz in*x*on*[a,∞]×Ω*, and*$\Omega \in R^{n}$*is a domain that contains the origin*x=0*. The constant*$x_{0}$*is an equilibrium point of Caputo fractional dynamic system (3), if and only is*$f\left(t,x_{0}\right)=0$.

**Lemma** **2.** *The Mittag–Leffler stability theorem [55]*

*If the equilibrium point of the nonlinear fractional dynamic system is*$x_{eq}=0$*, and D is the region containing the far point, then*$V(t,x(t)):[0,\infty )\times D\to R^{+}$ is a continuous differentiable function and satisfies

(4) $$\left\{ \begin{array}{l} V(t,x(t))\geq \gamma (\left\|x\right\|)\\ D^{\alpha }V(t,x(t))\leq 0 \end{array}\right.$$

*where* γ(⋅) *is a K class function. If* x∈D *and* 0<α<1*,* *then the equilibrium point* $x_{eq}=0$ is globally stable.

### 2.2. Description of the Two Fractional-Order Chaotic Systems

In order to improve the complexity of the classical three-dimensional fractional-order Lorenz system [56,57], two four-dimensional dissipative autonomous system models are constructed as Equations (5) and (6).

(5) $$\left\{ \begin{array}{l} \frac{d^{q}x_{1}}{dt^{q}}=20a_{1}x_{2}-27a_{1}x_{1}\\ \frac{d^{q}x_{2}}{dt^{q}}=7a_{1}x_{1}-x_{1}x_{3}+25a_{1}x_{2}\\ \frac{d^{q}x_{3}}{dt^{q}}=x_{1}x_{2}-3a_{1}x_{3}\\ \frac{d^{q}x_{4}}{dt^{q}}=0.1a_{1}x_{1}x_{3} \end{array}\right.$$

(6) $$\left\{ \begin{array}{l} \frac{d^{q}y_{1}}{dt^{q}}=35a_{2}y_{2}-20a_{2}y_{1}+5a_{2}y_{4}\\ \frac{d^{q}y_{2}}{dt^{q}}=7a_{2}y_{1}-y_{1}y_{3}+15a_{2}y_{2}\\ \frac{d^{q}y_{3}}{dt^{q}}=y_{1}y_{2}-3a_{2}y_{3}\\ \frac{d^{q}y_{4}}{dt^{q}}=0.1a_{2}y_{1}y_{3} \end{array}\right.$$

where $x_{1}$, $x_{2}$, $x_{3}$, $x_{4}$, $y_{1}$, $y_{2}$, $y_{3}$, and $y_{4}$ are state variables, q is the fractional order of the two systems (Equation (5) and Equation (6)), and $a_{1}$ and $a_{2}$ are parameters of the two systems. When 0<q<1 and $\;a_{1}=a_{2}=1$, the equilibrium of the system (5) is $O_{4}\left(0,0,0,w^{*}\right)$, where $w^{*}$ is an arbitrary real number, and the equilibrium of the system (6) is $O_{5}\left(0,0,0,0\right)$. So, the system (5) has countless equilibria, and the system (6) has only one equilibrium. The four eigenvalues of the Jacobian matrix at the equilibrium $O_{5}\left(0,0,0,0\right)$ are $\lambda_{1}=\;-25.9787$, $\lambda_{2}\;=\;20.9787$, $\lambda_{3}\;=\;3$, and $\lambda_{4}\;=\;0$. It is clear that equilibrium $O_{5}\left(0,0,0,0\right)$ is an unstable saddle-focus point. If both systems are dissipative autonomous systems, then the parameters must satisfy $\;a_{1}>0$ and $\;a_{2}>0$. 

### 2.3. Analysis of the Chaotic Behavior in the Two Fractional-Order Chaotic Systems

In order to study the nonlinear dynamic behavior of the two systems, numerical simulations were performed using the Matlab software (R2016a, MathWorks, Natick, MA, USA). The simulation algorithm uses the predictor–corrector scheme [58] to solve fractional differential equations. The initial values for the two systems were (1,2,2,3), the parameters were chosen to be q=0.8 and $\;a_{1}=a_{2}=1$, the simulation step size was set as h=0.01, and the number of simulation points *N* was 4000.

**Figure 1:**
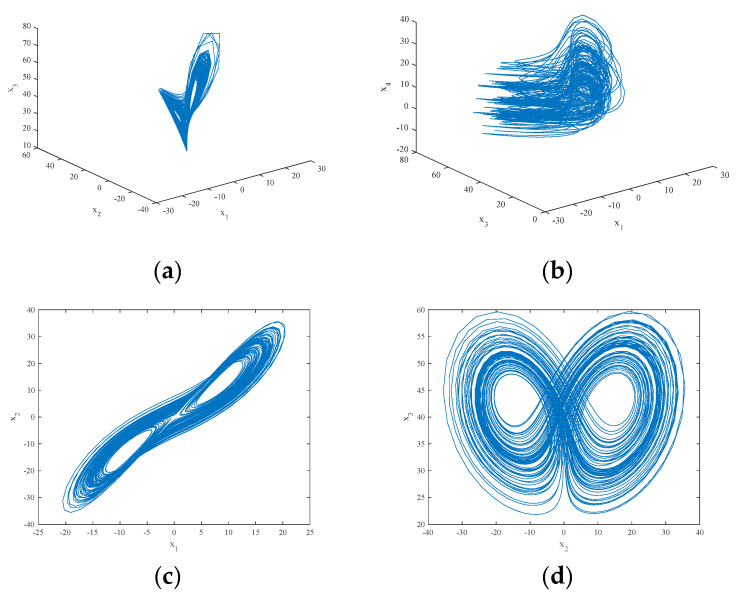
The projection of the chaotic attractor for the system (5): (**a**) in the $x_{1}-x_{2}-x_{3}$ space; (**b**) in the $x_{1}-x_{3}-x_{4}$ space; (**c**) on the $x_{1}-x_{2}$ plane; and (**d**) on the $x_{2}-x_{3}$ plane.

**Figure 2:**
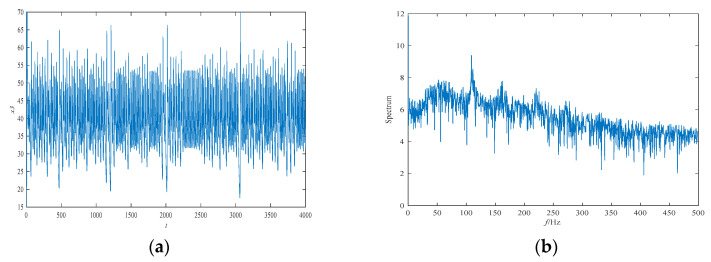
The time series of state variable $x_{3}$ and its frequency spectrum for system (5): (**a**) the time series; and (**b**) the frequency spectrum.

**Figure 3:**
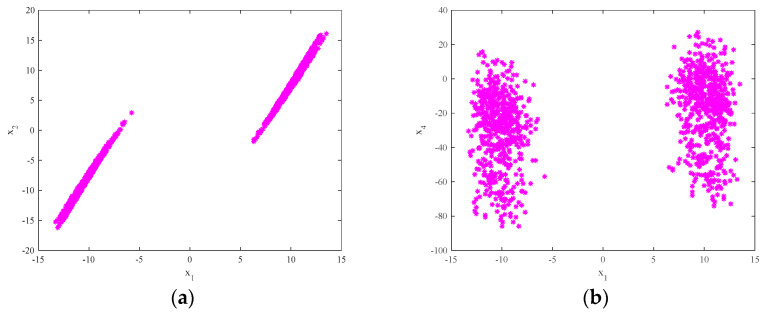
The Poincaré map of system (5)’s dependence on $x_{3}=50$: (**a**) the $x_{1}-x_{2}$ plane; (**b**) the $x_{1}-x_{4}$ plane.

The attractor projection of the new fractional-order chaotic system (5) is shown in Figure 1, which shows that the system motion’s trajectory is randomly separated into certain areas but is never closed. The system (5) is in a chaotic state and has a typical double-scroll chaotic attractor. At the same time, it can be seen from the time series of $x_{3}$ and its power spectral density, which was obtained by a Fourier transform of the autocorrelation function in Figure 2, that the system’s power spectrum is a nonperiodic continuous waveform, which is consistent with the characteristics of the random signals. Figure 3 plots the Poincaré map of the system (5)’s dependence on the plane of $x_{3}=50$, where the dense point set is shown. This means that the attractor has complex folding behavior and the dynamics of the system (5) are chaotic.

**Figure 4:**
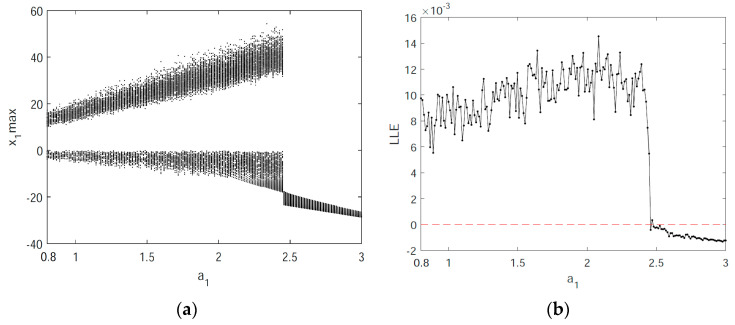
The bifurcation diagram and Largest Lyapunov exponents (LLEs) of the system (5) for $x_{1}$ with q=0.8 and $a_{1}\in (0.8,3)$: (**a**) the bifurcation diagram; and (**b**) the LLE graph.

**Figure 5:**
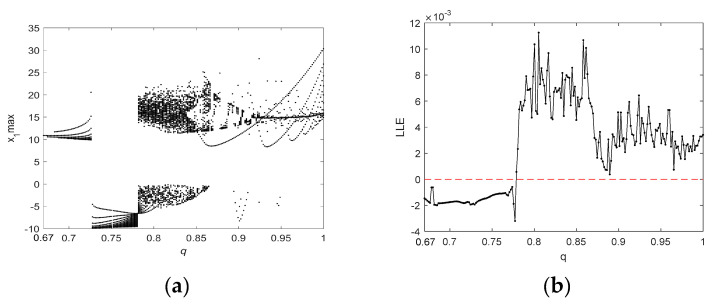
The bifurcation diagram and LLEs of the system (5) for $x_{1}$ with $a_{1}=1$ and q∈(0.67,1): (**a**) the bifurcation diagram; and (**b**) the LLE graph.

In order to further study the nonlinear chaotic behavior of the system (5), bifurcation diagrams and the Largest Lyapunov exponents (LLEs) of the system (5) are discussed below. Figure 4a depicts the bifurcation diagram of the state variable $x_{1}$ when making changes to the parameter $a_{1}$, and the corresponding LLE graph is shown in Figure 4b. In the $a_{1}\in (0.8,2.4)$ range, the LLE is positive, and the system is in a chaotic state. When $a_{1}\in (2.4,3)$, the LLE is negative, and the system is in a nonchaotic state. Additionally, the bifurcation diagram and LLE graph in Figure 5 illustrate the dynamic behavior of the system (5). Figure 5 exhibits the change in dynamic behavior of the system (5) for the state variable $x_{1}$ in the region of q∈(0.67,1). Specifically, when q∈(0.67,0.78), the bifurcation diagram shows that the system is in a nonchaotic state and has a negative LLE. When q∈(0.78,0.88), the bifurcation diagram shows that the system has transitioned from a periodic to a chaotic state and there is a positive LLE. When q∈(0.88,1), the LLE is a small positive number that is close to zero, and the bifurcation diagram illustrates different dynamic behaviors with lower complexity in the system, including weak chaotic and quasi-periodic limit cycle states.

**Figure 6:**
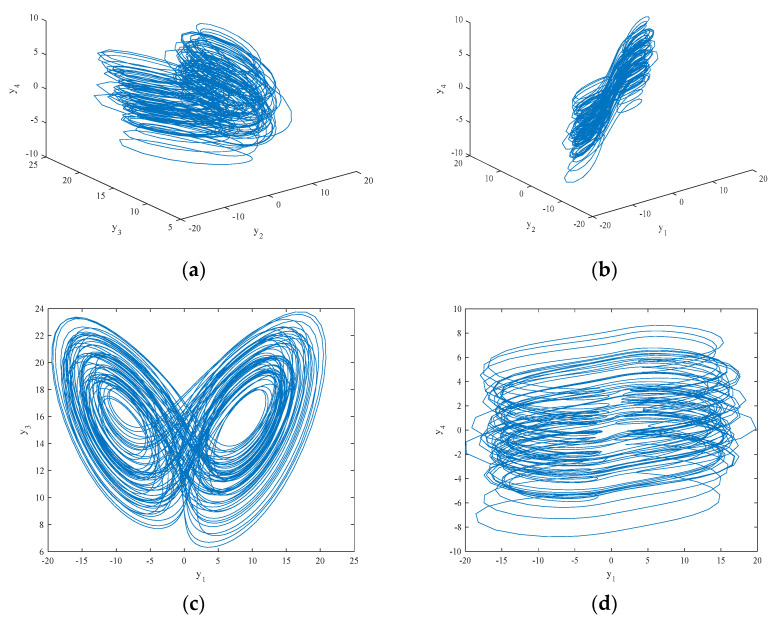
The projection of the chaotic attractor for the system (6): (**a**) in the $y_{2}-y_{3}-y_{4}$ space; (**b**) in the $y_{1}-y_{2}-y_{4}$ space; (**c**) on the $y_{1}-y_{3}$ plane; and (**d**) on the $y_{1}-y_{4}$ plane.

**Figure 7:**
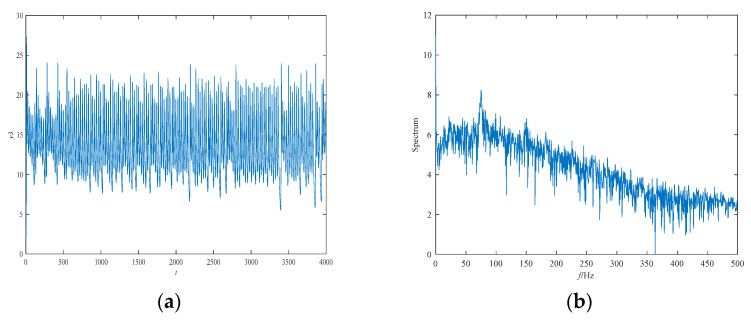
The time series of state variable $y_{3}$ and its frequency spectrum for system (6): (**a**) the time series; and (**b**) the frequency spectrum.

**Figure 8:**
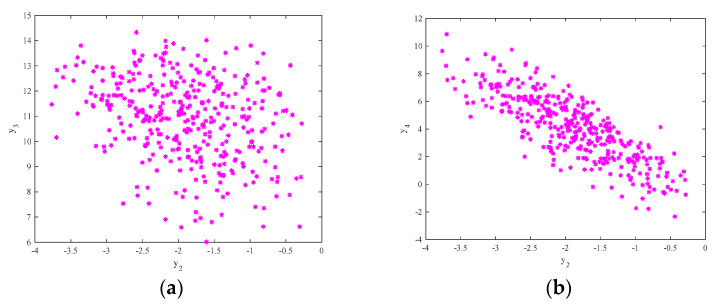
The Poincaré map of system (6)’s dependence on $y_{1}=0$: (**a**) the $y_{2}-y_{3}$ plane; and (**b**) the $\;y_{2}-y_{4}$ plane.

The attractor phase diagram of the new system (6) is presented in Figure 6. It can be seen from the three-dimensional and two-dimensional phase diagrams of the system that the system has a chaotic attractor with double scrolls. In addition, the time series of $y_{3}$ and its power spectral density are exhibited in Figure 7. The power spectral density is continuous and has no obvious peaks, and the time series aperiodic, which is consistent with the properties of chaotic signals. Finally, Figure 8 shows the Poincaré map of the system (6)’s dependence on $y_{1}=0$. There are dense point sets on the $y_{2}-y_{3}$ and $\;y_{2}-y_{4}$ planes, showing that the system has complicated folding behavior.

**Figure 9:**
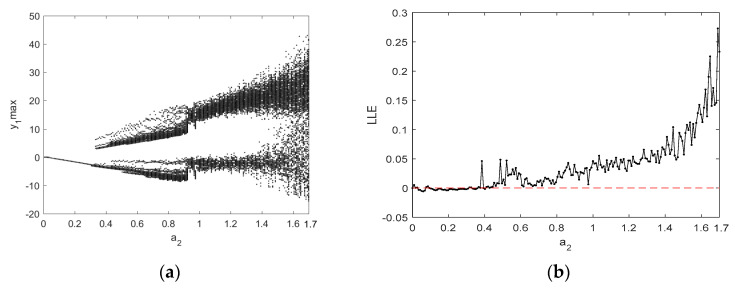
The bifurcation diagram and the LLEs of system (6) for $y_{1}$ with q=0.8 and $a_{2}\in (0,1.7)$: (**a**) the bifurcation diagram; and (**b**) the LLE graph.

**Figure 10:**
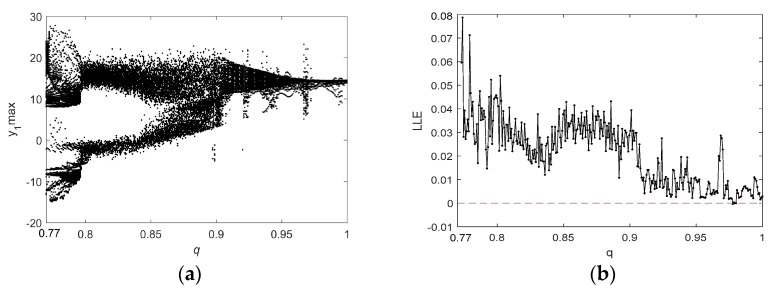
The bifurcation diagram and the LLEs of system (6) for $y_{1}$ with $a_{2}=1$ and q∈(0.77,1): (**a**) the bifurcation diagram; and (**b**) the LLE graph.

For q=0.8, the bifurcation diagram and the Largest Lyapunov exponents (LLE) of system (6) as a function of parameter $a_{2}$ are shown in Figure 9. Similarly, Figure 10 illustrates the dynamic chaotic behavior process in terms of a bifurcation diagram and an LLE graph when $a_{2}=1$ and as *q* increases from 0.77 to 1. Figure 9 indicates that, when $a_{2}\in (0,0.8)$, there are negative, zero, and small positive LLE values, and the system is in a periodic state or a weak chaotic state with low complexity. As the value of $a_{2}$ increases from 0.8, the LLE is positive, so system (6) has chaotic behavior. Figure 10a shows that the dynamic state of system (6) can be roughly divided into three phases. More specifically, in the range of q∈(0.77,0.8), the system exhibits complex bifurcation behavior, and there are large fluctuations in the corresponding LLE. When q∈(0.8,0.91), there is a positive LLE with small fluctuations and large values; hence, the system is in a chaotic state with high complexity. For the range of q∈(0.91,1), there is extremely complex dynamic behavior, including nonchaotic states, chaotic states, and weak chaotic states.

## 3. Entropy Analysis

As mentioned above, higher complexity implies higher security. In this section, we will further analyze the nonlinear dynamic behavior of the above two chaotic systems (system (5) and system (6)) with respect to the complexity of the time series. It is well-known that another statistical property of these systems is entropy, which is a measure of the dynamic chaotic behavior and has a certain relationship with the Lyapunov exponent and the Hausdorff dimension.

Generally, the complexity of chaotic systems is divided into behavior complexity and structural complexity. To date, several algorithms have been developed to calculate the complexity of a chaotic system’s behavior, and they all evolved from the Kolmogorov method and Shannon's entropy [59]. However, for chaotic systems with a high number of dimensions, the calculation results may overflow, leading to unexpected results. Structural complexity entails an analysis of the energy characteristics in a transformed domain, which means that the scope of analysis is holistic rather than local. Thus, the results obtained from a structural complexity analysis have more global meaning than the results obtained from a behavior complexity analysis [60]. For these reasons, research on structural complexity algorithms based on the Fourier transform and the wavelet transform, such as the spectral entropy algorithm (SE) and the small entropy algorithm, has made great progress. In addition, there is an improved complexity algorithm, called $C_{O}$, that is based on the fast Fourier transform (FFT). Due to its fast calculation speed, $C_{O}$ has many important properties and has achieved good complexity analysis results in practical applications. Moreover, since the signals in a real system are all analog, $C_{O}$ directly operates on continuous data without coarse-grain processing of the original data, thereby avoiding the changes in the dynamic property that may occur due to excessive coarse-grain processing. The $C_{O}$ algorithm can better describe the degree of randomness in system variables and more accurately describe the complexity of a chaotic system. Therefore, we adopted the $C_{O}$ algorithm to analyze the complexity of the system, and compared the results with those from the Lyapunov exponent and bifurcation analysis.

### 3.1. Description of the $C_{O}$ Complexity Algorithm

The $C_{O}$ complexity algorithm decomposes a sequence into regular and irregular components, reflecting the proportion of irregular components in the sequence. The steps for calculating the measured value are as follows.

Firstly, a discrete Fourier transformation is performed on the random sequence {x(n),n=0,1,2,3,⋯,M−1} for a given length by

(7) $$X(k)=\sum_{n=0}^{M-1}x(n)e^{-j\frac{2\pi nk}{M}}=\sum_{n=0}^{M-1}x(n)W_{M}^{nk}$$

where k=0,1,2,⋯,M−1.

Then, the mean square value of {X(k),k=0,1,2,3,⋯,M−1} is calculated by

(8) $$G_{M}=\frac{1}{M}{\sum_{k=0}^{M-1}\left|X(k)\right|}^{2}$$

Let

(9) $$\tilde{X}(k)=\left\{ \begin{array}{l} X(k)\begin{array}{cc} & if{\left|X(k)\right|}^{2}>rG_{M} \end{array}\\ 0\begin{array}{ccc} & & if{\left|X(k)\right|}^{2}\leq rG_{M} \end{array} \end{array}\right.$$

where r(r>0) is the control parameter. The inverse of the Fourier transform of $\tilde{X}(k)$ is performed using Equation (10).

(10) $$\tilde{x}(n)=\frac{1}{M}\sum_{k=0}^{M-1}\tilde{X}(k)e^{j\frac{2\pi }{M}nk}=\frac{1}{M}\sum_{k=0}^{M-1}\tilde{X}(k)W_{M}^{-nk}$$

where n=0,1,2,⋯,M−1. Finally, the $C_{O}$ algorithm’s complexity calculation is defined as in Equation (11).

(11) $$C_{O}(r,M)=\frac{\sum_{n=0}^{M-1}{\left|x(n)-\tilde{x}(n)\right|}^{2}}{\sum_{n=0}^{M-1}{\left|x(n)\right|}^{2}}$$

From the above process, we know that the larger the proportion of the energy in the irregular part in the sequence, the closer the corresponding signal is to the random sequence, and the greater the complexity. In addition, high-efficiency FFT and inverse fast Fourier transform (IFFT) techniques are used during processing, which makes the calculation speed of the $C_{O}$ algorithm very fast. In this paper, the $C_{O}$ complexity was used to analyze the dynamic behavior of the system, and the parameter r=15.

### 3.2. Influence of the Number of Simulation Points on Entropy

**Figure 11:**
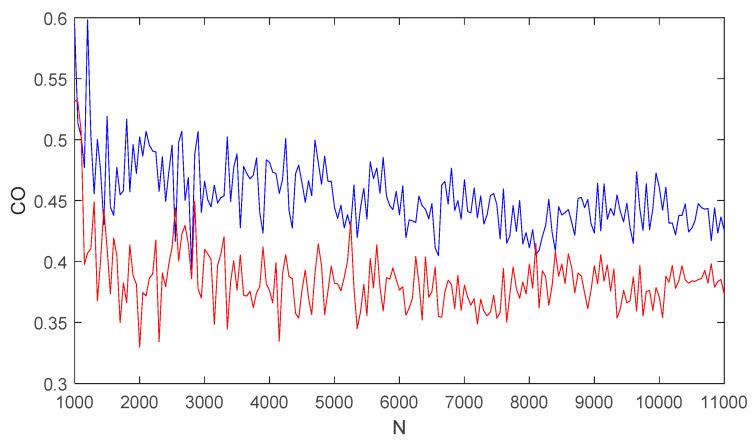
$C_{O}$ with respect to N for system (5) (Blue) and system (6) (Red).

For system (5) and system (6), the prediction–correction method [58] was used to solve the equation. For $a_{1}=a_{2}=1$, a simulation step size h=0.01, a fractional order q=0.8, and the initial conditions $\left(x_{1},x_{2},x_{3},x_{4}\right)=\left(y_{1},y_{2},y_{3},y_{4}\right)=(1,2,2,3)$, the $C_{O}$ complexity of the two systems with respect to the number of simulation points N is shown in Figure 11, where the blue curve corresponds to the $C_{O}$ of system (5) and the red graph corresponds to the $C_{O}$ of system (6). When N∈(1000,11000), Figure 11 shows that there are gentle fluctuations: the $C_{O}$ complexity of the two systems (5) and (6) fluctuates slightly around the two lines of $C_{O}=0.45$ and $C_{O}=0.38$, respectively. It is worth pointing out that as N increases the ripples become smaller, which indicates that the complexity of the system tends to be balanced for a large N value. Figure 11 also reflects that the value of $C_{O}$ for system (5) is larger than that of system (6); that is, system (5) is more complicated than system (6).

### 3.3. Influence of System Parameters on Entropy

**Figure 12:**
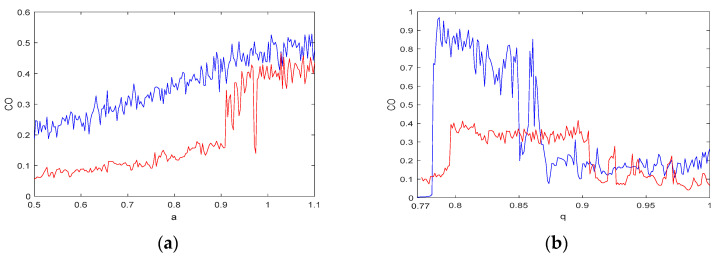
$C_{O}$ of system (5) (Blue) and system (6) (Red): (**a**) $C_{O}$ versus the parameters of the system; and (**b**) the $C_{O}$ complexity as a function of q.

There are three variables in systems (5) and (6): $a_{1}$, $a_{2}$, and q. For a simulation step size h=0.01 and N=4000, Figure 12 presents how the three variables affect the value of $C_{O}$. When fixing q=0.8, and varying the value of parameters $a_{1}$ and $a_{2}$ (where the coordinate variable a denotes $a_{1}$ and $a_{2}$, and a∈(0.5,1.1)), the blue curve represents the $C_{O}$ of $a_{1}$ for system (5) and the red graph represents the $C_{O}$ of $a_{2}$ for system (6), as shown in Figure 12a. The two curves indicate that the value of $C_{O}$ rises as a increases, and the complexity of the system (5) is greater than the complexity of the system (6), which is consistent with the results discussed in Section 3.2. Figure 12b shows the $C_{O}$ complexity as a function of q, where $a_{1}=a_{2}=1$ and q∈(0.77,1). As described in Section 2.3, there is chaotic behavior in both systems when q∈(0.8,0.85). The $C_{O}$ complexity has high values in this region as shown in Figure 12b, which means that the $C_{O}$ algorithm can better reflect the degree of dynamic behavior and the complexity in fractional-order chaotic systems. Moreover, Figure 12b also shows that the complexity of system (5) is greater than that of system (6), which is consistent with the previous results.

### 3.4. Analysis of the Chaotic Diagram of System Entropy

**Figure 13:**
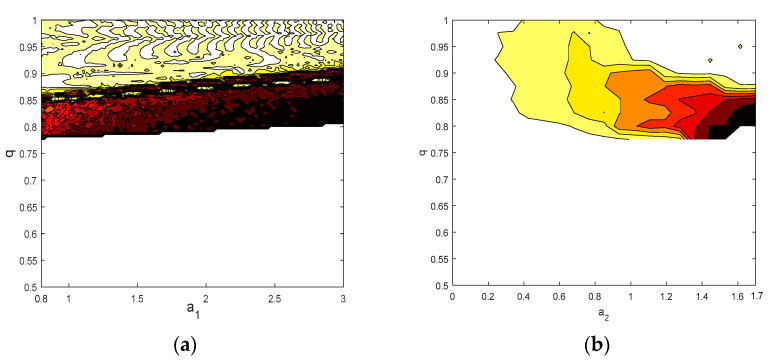
The chaotic characteristics of $C_{O}$ versus the system parameters: (**a**) the complexity on the $a_{1}-q$ plane; and (**b**) the complexity on the $a_{2}-q$ plane.

In order to reflect the distribution relationship between system parameters and $C_{O}$, a contour map with different color schemes was used to show the chaotic characteristics of the proposed systems. A simulation step size h=0.01, simulation points N=4000, and the prediction–correction method were used to solve the equations. Figure 13 describes the $C_{O}$ in terms of the system parameters $a_{1}$ and $a_{2}$ and the fractional order q. The contour map in Figure 13a shows that, in the range of q∈(0,0.78), system (5) is in a nonchaotic state. The high-complexity chaotic region is mainly concentrated in the range of q∈(0.78,0.88), which is consistent with the results of Figure 12b. As for the range of q∈(0.88,1), there are crisscross contours that represent different $C_{O}$ measurement values from the system. This also means that system (5) has multiple nonlinear dynamic behaviors, which also coincide with the bifurcation diagram and the maximum Lyapunov diagram of system (5) (shown in Figure 4 and Figure 5, respectively). For the $C_{O}$ complexity of system (6), it can be seen from Figure 13b that it is in accordance with the bifurcation diagram and the maximum Lyapunov diagram of system (6) (shown in Figure 9 and Figure 10, respectively). More specifically, when $a_{2}\in (0,0.21)$, the system is in a nonchaotic state; when $a_{2}\in (0.21,1.7)$, the system complexity will increase as q decreases. Throughout the whole of Figure 13, it can be observed graphically that when  q∈(0.77,0.85), the system complexity will increase as the value of $a_{1}$ and $a_{2}$ increases. This is consistent with the outcomes discussed in Section 3.3. This further confirms that the $C_{O}$ entropy method can be employed to measure the complexity of fractional-order chaotic systems, and provides an alternative method for the dynamic analysis of nonlinear systems in practical applications.

## 4. Adaptive Synchronization of Fractional-Order Systems

Based on the Mittag–Leffler stability theory, an adaptive control scheme will be developed to synchronize two dissipative autonomous systems: the drive system (5) and the response system (6). Results from numerical simulations and details of the process for the mathematical proof are presented to demonstrate the effectiveness of the proposed method.

### 4.1. Adaptive Synchronization for Determined Parameters

To analyze the synchronization, let us define the state errors between the driving system (5) and the response system (6). Then, the error system can be represented by

(12) $$\left\{ \begin{array}{l} e_{1}=y_{1}-x_{1}\\ e_{2}=y_{2}-x_{2}\\ e_{3}=y_{3}-x_{3}\\ e_{4}=y_{4}-x_{4} \end{array}\right.$$

The fractional-order error system (13) can be expressed as

(13) $$\left\{ \begin{array}{l} \frac{d^{q}e_{1}}{dt^{q}}=-27a_{1}e_{1}+35a_{2}e_{2}+y_{1}(27a_{1}-20a_{2})+x_{2}(35a_{2}-20a_{1})+5a_{2}y_{4}+u_{1}(t)\\ \frac{d^{q}e_{2}}{dt^{q}}=25a_{1}e_{2}+7a_{1}e_{1}+7y_{1}(a_{2}-a_{1})+y_{2}(15a_{2}-25a_{1})-e_{1}y_{3}-e_{3}x_{1}+u_{2}(t)\\ \frac{d^{q}e_{3}}{dt^{q}}=-3a_{1}e_{3}+3y_{3}(a_{1}-a_{2})+e_{1}y_{2}+e_{2}x_{1}+u_{3}(t)\\ \frac{d^{q}e_{4}}{dt^{q}}=0.1(a_{2}y_{1}y_{3}-a_{1}x_{1}x_{3})+u_{4}(t) \end{array}\right.$$

where $u_{1}(t)$, $u_{2}(t)$, $u_{3}(t)$, and $u_{4}(t)$ are error control functions, and $a_{1}$ and $a_{2}$ are the parameters of the driving system and the response system, which satisfy the conditions $a_{1}>0$ and $a_{2}>0$.

**Theorem** **1.** *For the drive system (5) and the response system (6), if the control function of the system is selected as follows*

(14) $$\left\{ \begin{array}{l} u_{1}(t)=27\hat{a}e_{1}-35a_{2}e_{2}-y_{1}(27a_{1}-20a_{2})-x_{2}(35a_{2}-20a_{1})-5a_{2}y_{4}\\ u_{2}(t)=-25\hat{a}e_{2}-7a_{1}e_{1}-7y_{1}(a_{2}-a_{1})-y_{2}(15a_{2}-25a_{1})+e_{1}y_{3}+e_{3}x_{1}\\ u_{3}(t)=3\hat{a}e_{3}-3y_{3}(a_{1}-a_{2})-e_{1}y_{2}-e_{2}x_{1}\\ u_{4}(t)=(\hat{a}-a_{1})e_{4}-0.1(a_{2}y_{1}y_{3}-a_{1}x_{1}x_{3}) \end{array}\right.$$

*where parameter* $\hat{a}$ *is an estimation of parameter* $a_{1}$ *, then the adaptive law of the estimated parameter is*

(15) $$D_{}^{q}\hat{a}=-27e_{1}{}^{2}+25e_{2}{}^{2}-3e_{3}{}^{2}-e_{4}{}^{2}+\lambda (\hat{a}-a_{1})$$

*If the parameter*λ≤0*, then the state error system has equilibrium*e=0*and*$a_{1}=\hat{a}$, the error system is globally asymptotically stable, thus, the response system is globally asymptotically synchronized with the drive system i.e., for any initial value, $\underset{t\to \infty }{\lim}\left\|e(t)\right\|=0$.

**Proof of Theorem** **1.** Substituting Equation (14) into Equation (13), one can obtain

(16) $$\left\{ \begin{array}{l} \frac{d^{q}e_{1}}{dt^{q}}=27e_{1}(\hat{a}-a_{1})\\ \frac{d^{q}e_{2}}{dt^{q}}=-25e_{2}(\hat{a}-a_{1})\\ \frac{d^{q}e_{3}}{dt^{q}}=3e_{3}(\hat{a}-a_{1})\\ \frac{d^{q}e_{4}}{dt^{q}}=e_{4}(\hat{a}-a_{1}) \end{array}\right.$$

It can be seen that the error dynamics system (16) appears to be independent of the response system parameter $a_{2}$. As long as the error system is stable, it is possible to synchronize two different chaotic systems with different parameters. According to Lemma 2 of the Mittag–Leffler stability theory, we construct the Lyapunov control function (17) by taking $e_{1}$, $e_{2}$, $e_{3}$, $e_{4}$, and $\hat{a}-a_{1}$ as variables.

(17) $$V(e,\gamma )=\frac{1}{2}e^{T}e+\frac{1}{2}\gamma^{2}$$

where $e={\left[e_{1},e_{2},e_{3},e_{4}\right]}^{T}$, $\gamma =\hat{a}-a_{1}$, and parameter $\hat{a}$ is an estimate of parameter $a_{1}$. The adaptive law of the estimated parameters is shown in Equation (15).

On the basis of Lemma 1, the derivative of the function (17) is

(18) $$\begin{array}{l} D_{}^{q}V\leq e_{1}\cdot D_{}^{q}e_{1}+e_{2}\cdot D_{}^{q}e_{2}+e_{3}\cdot D_{}^{q}e_{3}+e_{4}\cdot D_{}^{q}e_{4}+(\hat{a}-a_{1})\cdot D_{}^{q}\hat{a}\\ =27e_{1}{}^{2}(\hat{a}-a_{1})-25e_{2}{}^{2}(\hat{a}-a_{1})+3e_{3}{}^{2}(\hat{a}-a_{1})\\ \begin{array}{cc} & +e_{4}{}^{2}(\hat{a}-a_{1})+[-27e_{1}{}^{2}+25e_{2}{}^{2}-3e_{3}{}^{2}-e_{4}{}^{2}+\lambda (\hat{a}-a_{1})](\hat{a}-a_{1}) \end{array}\\ =\lambda {\left(\hat{a}-a_{1}\right)}^{2} \end{array}$$

According to the stability theory of Lemma 2, when λ≤0, then $D_{}^{q}V\leq 0$, there exists an equilibrium e=0 and $a_{1}=\hat{a}$; and the error system (16) is globally asymptotically stable, thus, the drive system (5) and response system (6) are globally asymptotically synchronized. This completes the proof. □

### 4.2. Adaptive Synchronization for Uncertain Parameters

For the situation of uncertain parameters, the drive system is taken to be Equation (19) and the response system is taken to be Equation (20).

(19) $$\left\{ \begin{array}{l} \frac{d^{q}x_{1}}{dt^{q}}=20ax_{2}-27ax_{1}\\ \frac{d^{q}x_{2}}{dt^{q}}=7ax_{1}-x_{1}x_{3}+25ax_{2}\\ \frac{d^{q}x_{3}}{dt^{q}}=x_{1}x_{2}-3ax_{3}\\ \frac{d^{q}x_{4}}{dt^{q}}=0.1ax_{1}x_{3} \end{array}\right.$$

(20) $$\left\{ \begin{array}{l} \frac{d^{q}y_{1}}{dt^{q}}=35ay_{2}-20ay_{1}+5ay_{4}+v_{1}(t)\\ \frac{d^{q}y_{2}}{dt^{q}}=7ay_{1}-y_{1}y_{3}+15ay_{2}+v_{2}(t)\\ \frac{d^{q}y_{3}}{dt^{q}}=y_{1}y_{2}-3ay_{3}+v_{3}(t)\\ \frac{d^{q}y_{4}}{dt^{q}}=0.1ay_{1}y_{3}+v_{4}(t) \end{array}\right.$$

where $v(t)={\left[v_{1}(t),v_{2}(t),v_{3}(t),v_{4}(t)\right]}^{T}$ is the control variable, and a is an unknown parameter. Assume that the system error function is

(21) $$\left\{ \begin{array}{l} e_{1}=y_{1}-x_{1}\\ e_{2}=y_{2}-x_{2}\\ e_{3}=y_{3}-x_{3}\\ e_{4}=y_{4}-x_{4} \end{array}\right.$$

Then, the fractional-order error system is

(22) $$\left\{ \begin{array}{l} \frac{d^{q}e_{1}}{dt^{q}}=-20a(e_{1}-e_{2})+15ay_{2}+7ax_{1}+5ay_{4}+v_{1}(t)\\ \frac{d^{q}e_{2}}{dt^{q}}=(7e_{1}+15e_{2})a-e_{1}y_{3}-e_{3}x_{1}-10x_{2}+v_{2}(t)\\ \frac{d^{q}e_{3}}{dt^{q}}=-3ae_{3}+e_{1}y_{2}+e_{2}x_{1}+v_{3}(t)\\ \frac{d^{q}e_{4}}{dt^{q}}=0.1a(e_{1}y_{3}+e_{3}x_{1})+v_{4}(t) \end{array}\right.$$

**Theorem** **2.** *For the drive system (19) and the response system (20), if the control function of the system is selected as*

(23) $$\left\{ \begin{array}{l} v_{1}(t)=20(e_{1}-e_{2})\hat{a}-15ay_{2}-7ax_{1}-5ay_{4}\\ v_{2}(t)=-7ae_{1}-35ae_{2}+e_{1}y_{3}+e_{3}x_{1}+10x_{2}\\ v_{3}(t)=-e_{1}y_{2}-e_{2}x_{1}\\ v_{4}(t)=-0.1(e_{1}y_{3}+e_{3}x_{1})a-e_{4}a \end{array}\right.$$

*where* $\hat{a}$ *is an estimate of parameter a, then the adaptive law for estimating the parameter is*

(24) $$D_{}^{q}\hat{a}=-20e_{1}{}^{2}+20e_{1}e_{2}$$

*The error system exists an equilibrium* e=0 *and* $a=\hat{a}$ *, the error system is globally asymptotically stable, thus, the response system is synchronized with the drive system globally and asymptotically, i.e.,* $\underset{t\to \infty }{\lim}\left\|e(t)\right\|=0$ *for any initial value.*

**Proof of Theorem** **2.** Substitute the control function (23) into the error system (22) and arrange the expressions as

(25) $$\left\{ \begin{array}{l} \frac{d^{q}e_{1}}{dt^{q}}=20(e_{1}-e_{2})(\hat{a}-a)\\ \frac{d^{q}e_{2}}{dt^{q}}=-20e_{2}a\\ \frac{d^{q}e_{3}}{dt^{q}}=-3e_{3}a\\ \frac{d^{q}e_{4}}{dt^{q}}=-e_{4}a \end{array}\right.$$

In order to synchronize two different chaotic systems with two unknown parameters, what we need to do is to stabilize the error system. In light of Lemma 2 of the Mittag–Leffler stability theory, $e_{1}$, $e_{2}$, $e_{3}$, $e_{4}$, and $\hat{a}-a_{1}$ are chosen as the variables for the Lyapunov control function (26).

(26) $$\left\{ \begin{array}{l} \frac{d^{q}e_{1}}{dt^{q}}=20(e_{1}-e_{2})(\hat{a}-a)\\ \frac{d^{q}e_{2}}{dt^{q}}=-20e_{2}a\\ \frac{d^{q}e_{3}}{dt^{q}}=-3e_{3}a\\ \frac{d^{q}e_{4}}{dt^{q}}=-e_{4}a \end{array}\right.$$

where $e={\left[e_{1},e_{2},e_{3},e_{4}\right]}^{T}$, $\gamma =\hat{a}-a$, and $\hat{a}$ is an estimate of parameter a. The adaptive law for estimating the parameters is shown in the Equation (24).

According to Lemma 1, the derivative of the Lyapunov function (26) is

(27) $$\begin{array}{l} D_{}^{q}V\leq e_{1}\cdot D_{}^{q}e_{1}+e_{2}\cdot D_{}^{q}e_{2}+e_{3}\cdot D_{}^{q}e_{3}+e_{4}\cdot D_{}^{q}e_{4}+D_{}^{q}\hat{a}\cdot (\hat{a}-a_{1})\\ =20e_{1}(e_{1}-e_{2})(\hat{a}-a)-20e_{2}{}^{2}a-3e_{3}{}^{2}a-e_{4}{}^{2}a+[-20e_{1}{}^{2}+20e_{1}e_{2}+\lambda (\hat{a}-a)](\hat{a}-a)\\ =-(20e_{2}{}^{2}+3e_{3}{}^{2}+e_{4}{}^{2})a+\lambda {\left(\hat{a}-a\right)}^{2} \end{array}$$

Since both the drive system (19) and the response system (20) are dissipative systems, it holds that a>0. Moreover, according to Lemma 2, there is an equilibrium e=0 and $a=\hat{a}$ when $D_{}^{q}V\leq 0$, i.e., $\underset{t\to \infty }{\lim}\left\|e(t)\right\|=0$. The error system (22) is globally asymptotically stable, thus, the drive system (19) and the response system (20) are globally and asymptotically synchronized. The proof is completed. □

### 4.3. Numerical Simulation

**Figure 14:**
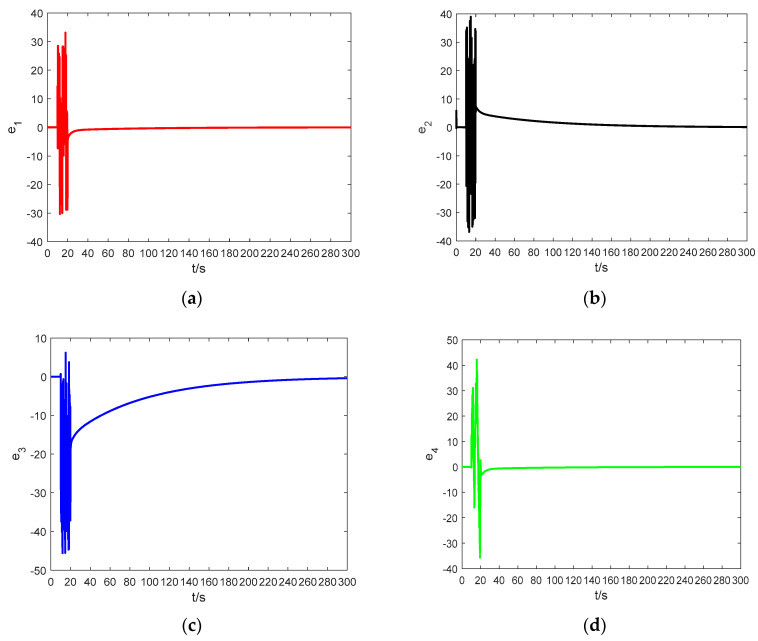
The synchronization errors between system (5) and system (6) based on Theorem 1: (**a**) the time response of $e_{1}$; (**b**) the time response of $e_{2}$; (**c**) the time response of $e_{3}$; and (**d**) the time response of $e_{4}$.

**Figure 15:**
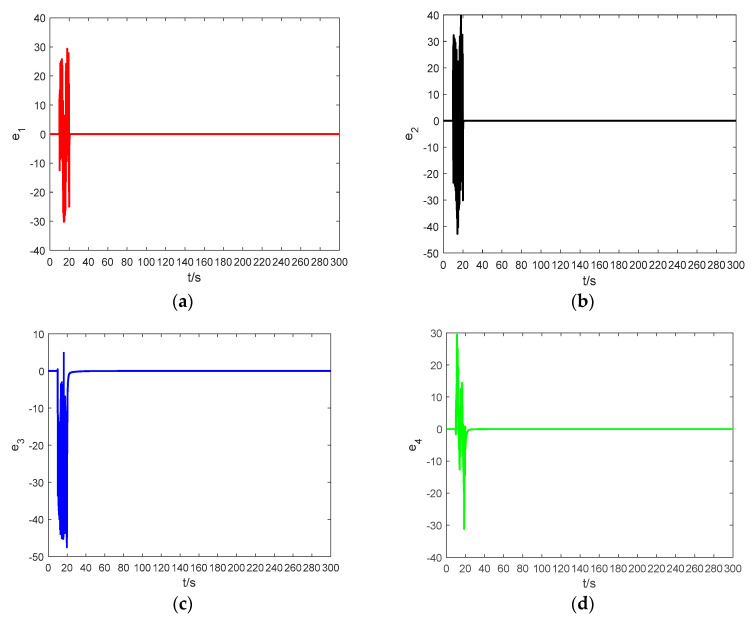
The synchronization errors between system (20) and system (21) based on Theorem 2: (**a**) the time response of $e_{1}$; (**b**) the time response of $e_{2}$; (**c**) the time response of $e_{3}$; and (**d**) the time response of $e_{4}$.

In order to demonstrate the effectiveness of the proposed method, a dynamic modeling simulation was performed using MATLAB R2016a. The simulation parameters were set as follows: the total simulation time is 300 s, an absolute tolerance and a relative tolerance of $10^{-3}$, λ=−1000, the solver adopted ode23tb(stiff/TR-BDF2), the initial value of the adaptive law was $\hat{a}(0)=0.5$, a fractional order q=0.8. Figure 14 shows the error graphs corresponding to the Theorem 1 when $a_{1}=1$ and $a_{2}=1.2$. Figure 15 shows the error curves corresponding to Theorem 2 for $a=a_{1}=a_{2}=1$. At t=0, the drive system and response system are set to have the same initial condition of (1,2,2,3).

During the period of 0≤t≤10 s, the controller is work. Figure 14 and Figure 15 demonstrate that the synchronization time is very short if the two systems have the same initial condition. When 10≤t≤20 s, the controller does not work. At t=20 s, the drive system and the response system are having arbitrary random values, and we let the controller work again. It can be observed that when 20≤t≤300 s, in spite of having different initial conditions, the synchronization between the drive system and the response system is also ideal. The error systems converge asymptotically to zero, which means that the adaptive controller based on the fractional-order Mittag–Leffler stability theory is reliable and very effective.

## 5. Conclusions

Two novel fractional-order chaotic systems with one equilibrium state and an infinite number of equilibria are proposed in this paper. The nonlinear dynamic behavior of the two systems was analyzed from the perspectives of equilibrium, a time domain diagram, power density graphs, an attractor phase diagram, bifurcation diagrams, and Poincaré maps. Moreover, by analyzing the $C_{o}$ entropy of the two four-dimensional fractional-order chaotic systems, the complex and nonlinear dynamics of the two systems were further verified. Therefore, if the two novel fractional-order chaotic systems are applied in such fields as communication security and image encryption, the unpredictability of the communication system can be enhanced, and the reliability and security of the system can be improved.

In addition, based on the Mittag–Leffler stability control theory, a fractional-order adaptive synchronization controller was designed, which could realize the decay of errors toward zero between two different fractional-order chaotic systems. The synchronization controller was investigated theoretically, and then dynamic modeling simulations were presented to verify the theoretical analysis. The adaptive controller designed in this paper has the following advantages. Firstly, the object of control that this method targets is a fractional-order chaotic system, which increases the complexity of the chaotic system. Secondly, adaptive synchronization scheme for both the determined parameters and the uncertain parameters is realized between different fractional-order chaotic systems, and an ideal synchronization effect was achieved. Finally, to avoid the process of constructing complex functions due to the use of the Lyapunov stability theory, the adaptive law was directly established by applying the Mittag–Leffler fractional-order stability theory. This synchronization strategy greatly simplifies the synchronization method for fractional-order chaotic systems, and the process for the proof of the method shows that the method is universal and practical. So, this paper provides a new theoretical method for the synchronization of fractional-order chaotic systems.
